# Deep Learning-Based Water-Fat Separation from Dual-Echo Chemical Shift-Encoded Imaging

**DOI:** 10.3390/bioengineering9100579

**Published:** 2022-10-19

**Authors:** Yan Wu, Marcus Alley, Zhitao Li, Keshav Datta, Zhifei Wen, Christopher Sandino, Ali Syed, Hongyi Ren, Lei Xing, Michael Lustig, John Pauly, Shreyas Vasanawala

**Affiliations:** 1 Radiology Department, Stanford University, Stanford, CA 94305, USA; 2 Hoag Hospital, Newport Beach, CA 92663, USA; 3 Electrical Engineering Department, Stanford University, Stanford, CA 94305, USA; 4 Radiation Oncology Department, Stanford University, Stanford, CA 94305, USA; 5 Electrical Engineering and Computer Science Department, University of California, Berkeley, CA 94720, USA

**Keywords:** dual-echo water-fat separation, deep learning, metal artifact mitigation

## Abstract

Conventional water–fat separation approaches suffer long computational times and are prone to water/fat swaps. To solve these problems, we propose a deep learning-based dual-echo water–fat separation method. With IRB approval, raw data from 68 pediatric clinically indicated dual echo scans were analyzed, corresponding to 19382 contrast-enhanced images. A densely connected hierarchical convolutional network was constructed, in which dual-echo images and corresponding echo times were used as input and water/fat images obtained using the projected power method were regarded as references. Models were trained and tested using knee images with 8-fold cross validation and validated on out-of-distribution data from the ankle, foot, and arm. Using the proposed method, the average computational time for a volumetric dataset with ~400 slices was reduced from 10 min to under one minute. High fidelity was achieved (correlation coefficient of 0.9969, $l_{1}$ error of 0.0381, SSIM of 0.9740, pSNR of 58.6876) and water/fat swaps were mitigated. I is of particular interest that metal artifacts were substantially reduced, even when the training set contained no images with metallic implants. Using the models trained with only contrast-enhanced images, water/fat images were predicted from non-contrast-enhanced images with high fidelity. The proposed water–fat separation method has been demonstrated to be fast, robust, and has the added capability to compensate for metal artifacts.

## 1. Introduction

Since the invention of the Dixon method, chemical shift-encoded images have been used to produce water and fat images based on the distinct in-phase/out-of-phase cycling of fat and water [1,2]. While water and fat images can be obtained from three or more chemical shift-encoded images, dual-echo water–fat separation is more commonly used clinically (as the in-phase and opposed-phase sequence has become an essential part of the routine MRI protocols of many anatomic regions [3]). In particular, in contrast to enhanced imaging (which is critical for cancer management), dual-echo water–fat separation is predominantly used because of its speed advantage over triple-echo imaging.

In chemical shift-encoded water–fat separation, a major challenge is to estimate the phase error caused by magnetic field inhomogeneity. This is essentially a nonlinear parameter estimation problem that is complicated by several practical factors, such as ambiguities in the signal model, the presence of low-signal regions, and the rapid spatial variation of $B_{0}$ homogeneity (especially when it is near metal [4]). Various optimization methods have been proposed in the past twenty years. These methods impose a smoothness on the magnetic field map and include techniques such as region growing, regional iterative phasor extraction, graph cut, and tree-reweighted message-passing algorithms [5,6,7,8,9,10,11,12,13,14,15,16,17,18]. Despite the impressive progress, water/fat swaps (which are caused by inaccurate phase estimation due to $B_{0}$ inhomogeneity) have not been completely eliminated and are still observed in daily clinical practice, especially when high spatial resolution acquisitions force a significant deviation of echo times from the optimal in-phase and opposed-phase times. Moreover, robust state-of-the-art separation algorithms that mitigate these issues suffer long computational times.

For dual-echo water–fat separation, the recently developed projected power method has proven to be a relatively fast and reliable quadratic optimization approach [18]. It can be viewed an extension of the VARPRO algorithm [4], since they both use similar spatial weighting schemes to impose field map smoothness in the setting of dual-echo water–fat separation. By offering relatively high robustness and high computational efficiency (as compared to other optimization methods), the projected power method has attracted substantial research attention and has been incorporated into the clinical routine at our university hospitals. However, the computational time is still lengthy for widespread practical clinical use, especially for contrast-enhanced imaging (since technologists must rapidly evaluate the images for adequacy).

Hypothetically, deep learning [19] has great potential for improving $B_{0}$ estimation and water–fat separation due to its superior capability in incorporating *a priori* knowledge. For $B_{0}$ estimation, conventional water–fat separation approaches only depend on spatial smoothness of the magnetic field homogeneity. Using deep learning, a variety of *a priori* knowledge can be extracted from statistical/spatial perspectives and complicated nonlinear mapping can be realized with multiple contributing factors taken into consideration. This will be particularly useful in the regions where magnetic field strength changes dramatically (e.g., near metal).

Furthermore, a hierarchical deep neural network is expected to improve the robustness of water–fat separation by providing a large neighboring pixel set. In principle, a substantially large neighboring pixel set is needed to impose field map smoothness and avoid local water/fat swaps. However, the actual neighboring pixel set used in conventional methods is typically small to save computation time [18]. To mitigate the problem, multiresolution methods were proposed and proven to be effective [13]. Via a similar multiresolution scheme, a hierarchical deep network can offer a large receptive field that benefits robust water–fat separation.

In the past few years, deep learning has been used for water–fat separation from a large number of chemical shift-encoded images [20,21,22,23]. In a cardiac study, water/fat images and an $R_{2}{}^{*}$ map were obtained from twelve multi-echo images [20]. In whole-body MRI scans, water/fat images were derived from up to five multi-echo images [21]. In a knee study, water/fat images were extracted from six multi-echo images, where synthetic magnetic field inhomogeneities were applied to enlarge the training set [22]. In an abdominal study, a water–fat separation model was established based on eight multi-echo images, where the generalization capabilities (with various imaging parameter values, field inhomogeneities, and anatomic regions) were evaluated [23]. All of these deep learning-based water–fat separation methods require a large number of chemical shift-encoded images as input, which is impractical in clinical use.

In this study, we propose a deep learning approach to derive water and fat images from dual-echo images, aimed at achieving near-instant water–fat separation with high robustness and short scan times. Dual-echo imaging, which is widely used clinically, was used to minimize scan time. This offers a significant advantage over previous deep learning-based water–fat separation methods, which require many multi-echo images at the cost of long scan times. While deriving water/fat information from fewer input images is more challenging, here we aim to determine whether it is feasible. As will be shown, water/fat images predicted from dual-echo images using the proposed method not only have high fidelity to the reference images but also improve over the reference images in several aspects, including the mitigation of local and global water/fat swaps and the correction of artifacts induced by metallic implants.

In addition, the generalization of the capabilities of the proposed method were investigated. We tested out-of-distribution data (from the ankle, foot, and arm) using deep learning models trained with only knee images. We evaluated special cases that had metal implants, where the training set of the prediction model lacked any examples with metallic implants. We predicted water/fat images from non-contrast-enhanced images based on models established with only contrast-enhanced images and, additionally, where the input images were acquired using flexible imaging parameters. The proposed deep learning method demonstrated promising results in all these cases.

## 2. Method

**Figure 1:**
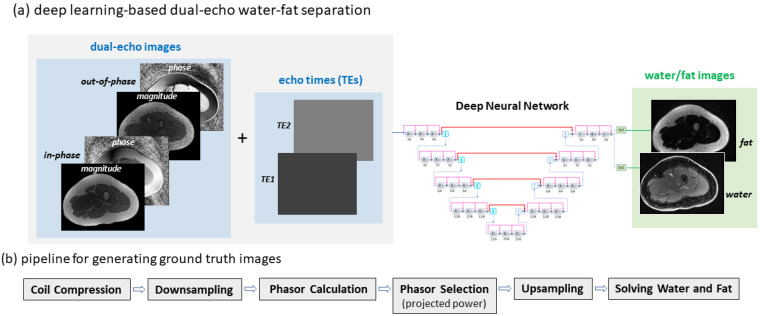

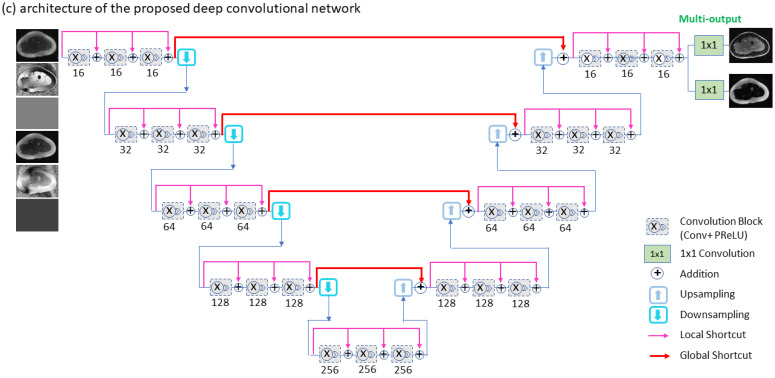
Deep learning-based water–fat separation from dual-echo images. (**a**) A deep neural network was employed to provide end-to-end mapping from dual-echo images and related echo times to the corresponding water/fat images. (**b**) Clinical pipeline for generating reference water/fat images from dual-echo images using the projected power method. Here, phasor calculation and phasor selection were the only components used for water–fat separation, while coil compression and downsampling/upsampling were used to reduce data processing time. (**c**) A multi-output deep convolutional neural network for water–fat separation had a hierarchical network architecture and unique shortcut connections.

A densely connected hierarchical convolutional network was employed to provide end-to-end mapping from dual-echo images to the corresponding water/fat images. Here, the ground truth or reference water/fat images were obtained using the projected power approach [18], which offered relatively high robustness and short postprocessing time (as compared to other optimization methods). As the input to the network, both magnitude and phase of in-phase and out-of-phase images were used. Moreover, the echo times used to acquire in-phase/out-of-phase images were included as an additional input to provide full support for the use of flexible imaging parameters. The approach is illustrated in Figure 1a.

### 2.1. Data Acquisition and Image Reconstruction

With IRB approval and informed patient consent, raw data from 68 pediatric clinically indicated dual echo scans were analyzed, corresponding to 19,382 contrast-enhanced images (patients had tumors in the knee, ankle, foot, or arm; 33 males and 35 females; a median age of 11 years, ranging from 15 months to 21 years old). In addition, non-contrast-enhanced dual-echo images were acquired from a healthy volunteer (male, 45 years old). The data sets were acquired on various GE MRI scanners (GE Healthcare, Waukesha, WI, USA), including two 3T MR750 scanners, a 3T PET-MRI scanner, and a 1.5T GE Artist scanner.

For dual-echo contrast-enhanced imaging, a 3D spoiled-gradient echo sequence was applied with variable density Poisson disc sampling pattern [24], achieving a net acceleration factor of 1.5. A 16-channel GEM Flex coil or a 32-channel cardiac coil was used. Based upon prescribed image resolution and system gradient strength, two clusters of TR values (4.48–4.78 ms or 6.54–7.39 ms) were applied for data acquisition at 3T. The corresponding TE values were 2.23 ms for in-phase images, while two clusters (1.21–1.31 ms or 3.35 ms) were used for out-of-phase images. For the images obtained at 1.5T, the TEs for in-phase and out-of-phase images were 4.17 and 2.08 ms, respectively. Other imaging parameters were as follows: bandwidth = 192 kHz; FOV = 32 × 36 cm; matrix size = 512 × 512; number of slices = 292–440; slice thickness = 1 mm; flip angle = 15; and scan time = 2 min 48 s–6 min 10 s for a 3D image volume. In addition, a non-contrast-enhanced examination was performed using the same sequence with different imaging parameter (an acceleration factor of 2, a bandwidth of 83.3 kHz, a flip angle of 25°, or a phase encoding of 224).

Dual-echo images were reconstructed with the ARC (Autocalibrating Reconstruction for Cartesian imaging) algorithm [25]. To reduce the reconstruction time, coil compression (from a 16-channel GEM Flex coil or a 32-channel cardiac coil into 6 virtual coils) was applied prior to parallel imaging reconstruction [26]. To preserve the phase between two echoes, the same coil compression matrices were applied to data from both echoes.

### 2.2. Generating Reference Water/Fat Images

In this study, reference water/fat images were produced from dual-echo images using the projected power approach [17].

In dual-echo imaging, the signal intensity of in-phase and out-of-phase images can be represented by

$$S_{1}=\left(W+C_{1}F\right)e^{i{Ø}_{1}},\;S_{2}=\left(W+C_{2}F\right)e^{i{Ø}_{2}},$$

where $S_{1}$, $S_{2}$, W, and F are in-phase, out-of-phase, water, and fat images; $C_{1}$ and $C_{2}$ are dephasing factors for fat with respect to water; and ${Ø}_{1}$ and ${Ø}_{2}$ are additional phases caused by $B_{0}$ inhomogeneity. Since the number of unknown variables is greater than the number of linear equations, the equations have multiple solutions for phasor P ($P=e^{iØ}$)

$$P_{1}=\frac{S_{1}*S_{2}}{\left(W_{1}+C_{1}*F_{1}\right)\left(W_{1}+C_{2}F_{1}\right)},\;P_{2}=\frac{S_{1}*S_{2}}{\left(W_{2}+C_{1}*F_{2}\right)\left(W_{2}+C_{2}F_{2}\right)},$$

and the water–fat separation problem reduces to choosing the right P from the two potential solutions. This can be further reformulated into a binary quadratic optimization problem as given by

$$minimize_{x}\;f\left(x\right)=X^{T}VX=\underset{r,s}{\sum }X{\left(r\right)}^{T}V\left(r,s\right)X\left(s\right)$$

$$subject\;to\;X_{r}\in \;\left[\left(\begin{array}{c} 1\\ 0 \end{array}\right),\left(\begin{array}{c} 0\\ 1 \end{array}\right)\right]\;,\;\forall r$$

Here, X indicates the binary selection of the phasor candidates and V(r,s) encodes 4 possible values of V for a pixel pair (r,s)

$$V\left(r,s\right)=\{\begin{array}{c} \left[\begin{array}{c} V\left(P_{1}\left(r\right),P_{1}\left(s\right)\right),V\left(P_{1}\left(r\right),P_{2}\left(s\right)\right)\\ V\left(P_{2}\left(r\right),P_{1}\left(s\right)\right),\;V\left(P_{2}\left(r\right),P_{2}\left(s\right)\right) \end{array}\right]\;,\;if\;s\in N_{r}\\ 0,\;otherwise \end{array}$$

where V is a quadratic penalty that enforces field map smoothness

$$V\left(P\left(r\right),P\left(s\right)\right)=\frac{\min\left(\left|S_{1}\left(r\right)\right|,\;\left|S_{1}\left(s\right)\right|\right)}{d\left(r,s\right)}{\left|P\left(r\right)-P\left(s\right)\right|}^{2}$$

To solve the binary quadratic optimization problem, an iterative projected power method was employed with a neighboring pixel set of 56 empirically selected pixels [17]. In addition, to speed up data processing, coil compression was conducted (compressing data from a 16-channel GEM Flex coil or a 32-channel cardiac coil to 6 virtual channels, reducing data processing time by a factor of 6 or 14); downsampling was performed on the original images (from a resolution of ~0.6 × 0.6 × 1 ${\mathrm{mm}}^{3}$ to 6 × 6 × 6 ${\mathrm{mm}}^{3})$ to accelerate data processing by another factor of ~600; and upsampling was applied to generated water/fat images accordingly. The pipeline is illustrated in Figure 1b. After manual correction for global water/fat swaps, the resulting water/fat images were taken as reference images.

### 2.3. Separating Water and Fat Images Using Deep Learning

For dual-echo water–fat separation, a densely connected hierarchical convolutional network was employed. This is a variant of T-Net [27] with s modification for multi-output. Originally developed for MR image reconstruction, T-Net has demonstrated high performance in image-to-image translation tasks [28,29,30,31,32]. It has a hierarchical network architecture with global shortcuts (similar to U-Net [33]) and densely connected local shortcuts (inspired by [34,35,36]). Downsampling and upsampling are accomplished using convolution rather than max-pooling (suggested by [37]). More specifically, there are five hierarchical levels in the network. At each level, there are three convolutional blocks. A convolutional block at different levels has 16, 32, 64, 128, and 256 channels, respectively. Image features are extracted using 3 × 3 convolutional kernels, followed by a Parametric Rectified Linear Unit (PReLU). Downsampling and upsampling are accomplished using 2 × 2 convolutional kernels with a stride of 2. The network architecture is illustrated in Figure 1c.

A multi-output deep neural network was constructed to simultaneously derive water and fat images. To obtain both water and fat images, there are different network designs. One approach is to employ two subnets, each generating a specific type of image (water or fat) with a 1 × 1 kernel at the last layer. Another option is to use a single network that produces multiple outputs with several 1 × 1 kernels at the last layer. The multi-output network solution is more efficient, since a large number of features extracted from input images can be shared between the output water/fat images. For this reason, a multi-output network was employed in this study and implemented on NiftyNet [38], a tensor flow-based AI platform [39].

A unique design of the proposed deep learning method was to include imaging parameters as additional network input. For every slice, not only were dual-echo images used as input but also images that provide in-phase and out-of-phase echo times at every pixel (Figure 1a). While mapping from dual-echo images to water–fat images can be learned in the absence of TEs, explicit provision of this *a priori* knowledge is expected to improve the prediction accuracy, particularly when flexible imaging parameter values are adopted for data acquisition.

While we primarily used original images as input and reference, images segmented to remove non-tissue background were also used as input and reference to investigate whether the background noise in the input images impeded model training.

A total of 17,874 images from 59 knee patients were used for training and testing with eight-fold cross validation applied. Of all the images, fifty-four data sets were acquired on 3T MRI scanners, one data set was obtained on a 1.5T MRI scanner, and four data sets were collected on a 3T PET-MRI scanner. Two data sets had metallic implants. We excluded from the training data the 1.5T image set as well as 3T images that had severe water/fat swaps or metal artifacts. In every training set, we included images acquired with different clusters of out-of-phase TEs (i.e., some TEs were between 1.21–1.31 ms, while others were 3.35 ms).

In addition, out-of-distribution data from other anatomic regions were tested using the models trained with only knee images. This included 1010 images from 5 ankle/foot patients and 948 images from 4 arm patients.

Given models established with contrast-enhanced images, non-contrast-enhanced dual-echo images of the knee from a healthy volunteer were used to derive water/fat images. At every scan, the value of one imaging parameter (acceleration factor, bandwidth, flip angle, phase encoding) or $B_{0}$ shimming was changed so that the model’s capability to support flexible imaging parameters could be evaluated.

In training, network parameters were initialized using the He method [40]. The $l_{1}$ loss was calculated, backpropagated, and used to update network parameters using the Adam algorithm [41] with an adaptive learning rate starting from 0.001, $\beta_{1}$ of 0.9, $\beta_{2}$ of 0.999, and ϵ of $10^{-8}$.

For the predicted images, quantitative metrics of the correlation coefficient, $l_{1}$ error, Structural Similarity Index Measure (SSIM), and peak Signal-to-Noise Ratio (pSNR) were calculated. Notice that the quantitative evaluation revealed the fidelity of the predicted images to the reference images but not to the ground truth (the slight improvements in the predicted images were even taken as errors, whereas the slices with severe water/fat swaps or metal artifacts in reference images were excluded). A board-certified radiologist reviewed the reference/predicted images for water/fat swaps.

## 3. Results

On average, the data processing time required for a two-dimensional image was 0.13 s using an established deep learning model, as compared to 1.5 s using the projected power approach (which had been significantly accelerated with the downsampling of the input images). For the average volumetric dataset with ~400 slices, processing time was reduced from 10 min to under one minute in our university hospitals.

**Figure 2:**
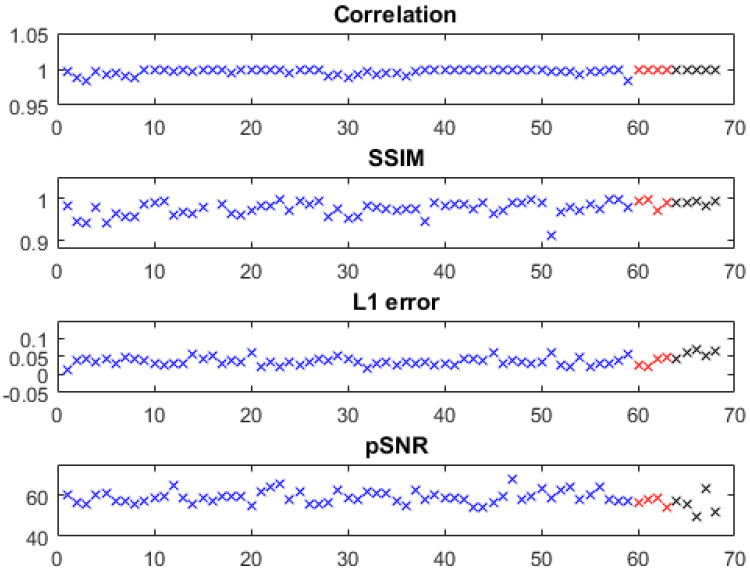
Quantitative evaluation on predicted water images. Metrics of the knee, arm, and foot/ankle images are marked with blue, red, and black, respectively. (1) Using the models trained with only knee images, water-fat separation was performed on knee images from 59 subjects, foot/ankle images from 5 subjects, and arm images from 4 subjects. Overall, the correlation coefficient was between 0.9833 and 1.0000 with mean/std of 0.9969 ± 0.0040, the $l_{1}$ error was between 0.0346 and 0.0648 with mean/std of 0.0381 ± 0.0122, the SSIM was between 0.9994 and 1.0000 with mean/std of 0.9740 ± 0.0207, and the pSNR was between 49.6049 and 67.5389 with mean/std of 58.6876 ± 3.2359. A subject was excluded from quantitative evaluation because most of the reference images had severe water/fat swaps (Figure 5b).

The quantitative metrics of the predicted water images of the knee, foot, ankle, and arm are shown in Figure 2, where slices with severe problems in reference images (e.g., Figures 5a and 6a,b) were excluded. Using models trained with only knee images, we obtained high fidelity in test images of the knee as well as in out-of-distribution data from the foot, ankle, and arm with a correlation coefficient of 0.9969 ± 0.0040, $l_{1}$ error of 0.0381 ± 0.0122, SSIM of 0.9740 ± 0.0207, and pSNR of 58.6876 ± 3.2359 (metrics of the knee, arm, and foot/ankle images are marked with blue, red, and black, respectively). The out-of-distribution data had very close accuracies to the knee images.

**Figure 3:**
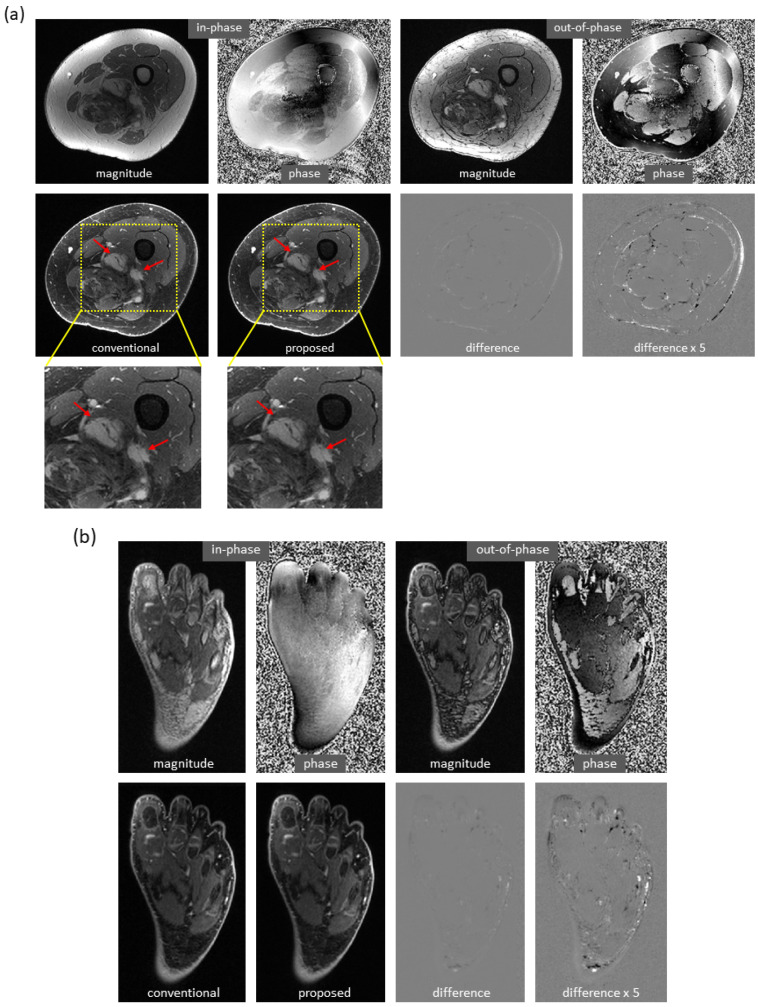
High fidelity achieved in dual-echo water–fat separation (only water images are shown). (**a**) Using a well-trained deep learning model, highly accurate water images of the knee were predicted from in-phase and out-of-phase images (as well as echo times, not shown in the figure). The enhancing tumors (arrows) were well delineated. Notably, this test set was acquired on a PET-MRI scanner. (**b**) Using a deep learning model trained with only knee images, the predicted image of the foot has achieved high fidelity to the reference image, despite inference on a different anatomic region.

Water and fat images derived using the proposed method have high fidelity to the reference images. An example of the knee is demonstrated in Figure 3a. The differences between the predicted and reference images were negligible. In the predicted image, the enhancing tumors were well delineated. It is of note that this test set was acquired on a PET-MRI scanner (whereas the majority of the training data were obtained on MRI scanners). The models trained with only knee images also work well on out-of-distribution data from the foot, ankle, and arm. An example of the foot is demonstrated in Figure 3b (here, the images had a high spatial resolution that is not routinely obtained with dual-echo gradient echo imaging).

**Figure 4:**
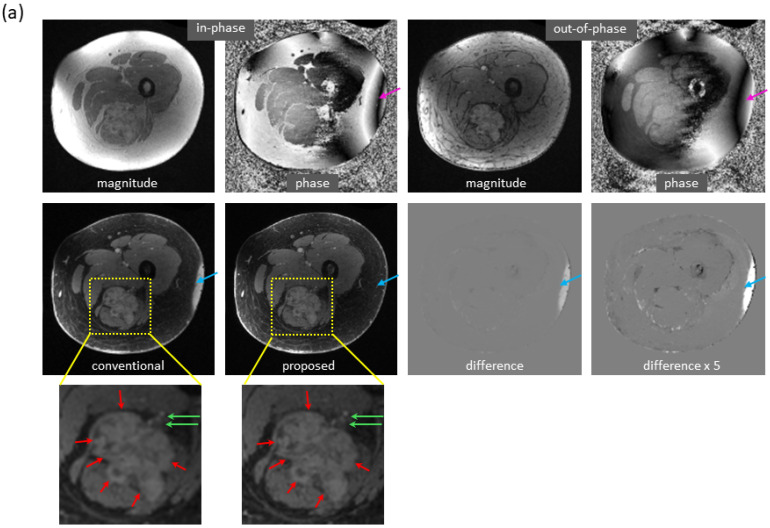

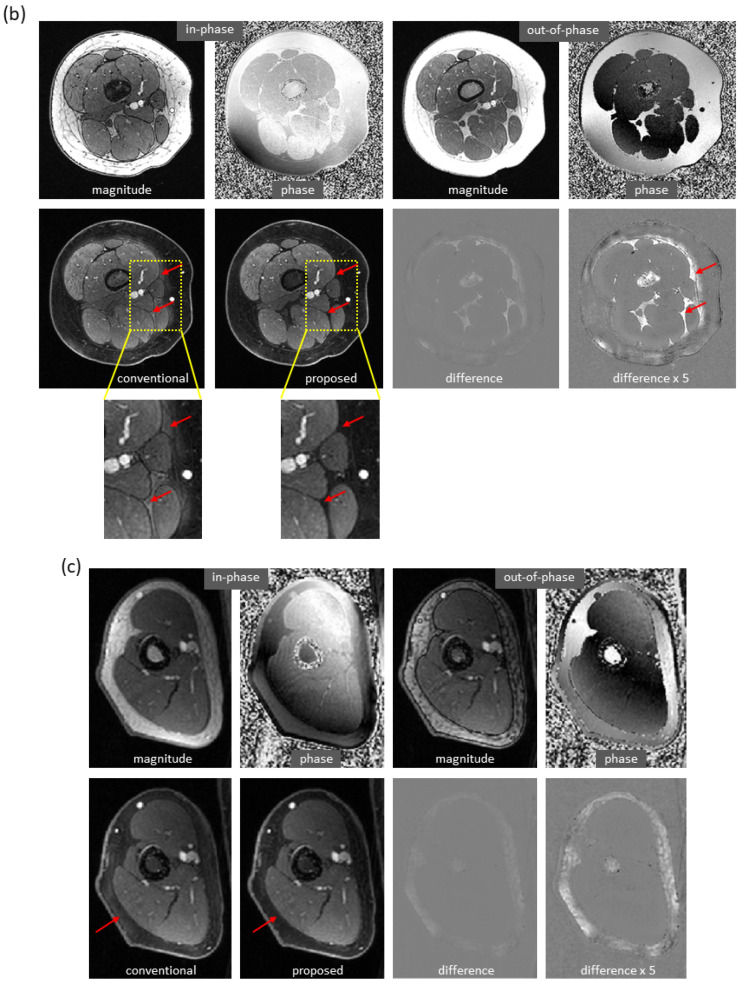

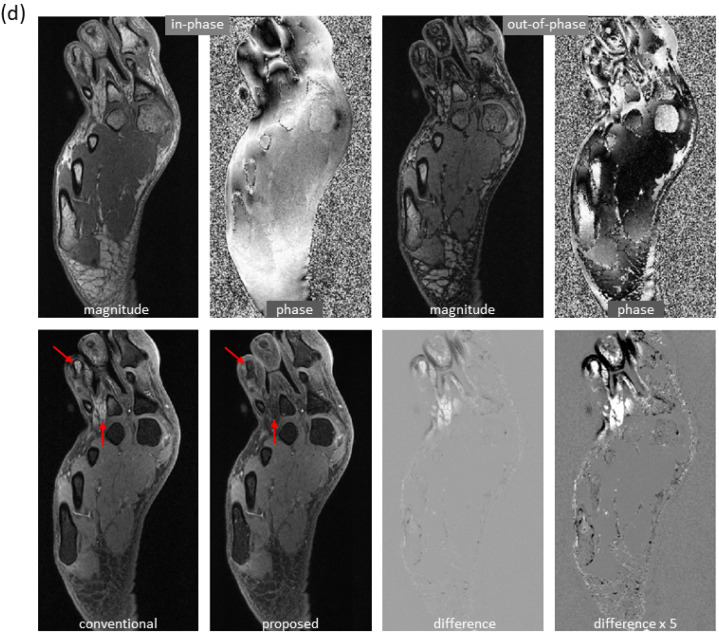
Mitigation of slight water/fat swaps. (**a**) Artifacts in an off-isocenter reference image of the knee (blue arrows), which were introduced by severe $B_{0}$ inhomogeneities, were automatically compensated in the predicted image. Here, tumor (red arrows) and adjacent nerve (green arrows) were well separated in the predicted image. (**b**) Water–fat separation was improved in the predicted image of the knee as compared to the reference image, which had regions of error (arrows). (**c**) More complete water–fat separation was observed in the predicted image of the arm, even if the deep learning model was trained with only knee images. (**d**) Some local water/fat swaps that appeared in the reference image of the foot did not occur in the predicted image. It is of note that the images (**c**,**d**) demonstrated the robustness of the approach to out-of-distribution data.

Using the proposed method, water/fat swaps that appeared in reference images were mitigated in the predicted images. Figure 4 demonstrates some examples with minor water/fat swaps. In Figure 4a, $B_{0}$ inhomogeneities (in a slice far away from the isocenter) led to artifacts in a reference image of the knee; the artifacts did not appear in the predicted image. Here, tumor and adjacent nerve were well delineated in the predicted image, which is critical for surgical planning. In Figure 4b,c, a more complete separation of water and fat signals was achieved in the predicted images of the knee and arm. In Figure 4d, local water/fat swaps in a reference image of the foot were largely compensated in the predicted image. Notably, these images of the arm and foot were inferred using models trained with only knee data, and the robustness of the proposed approach to out-of-distribution data was demonstrated.

**Figure 5:**
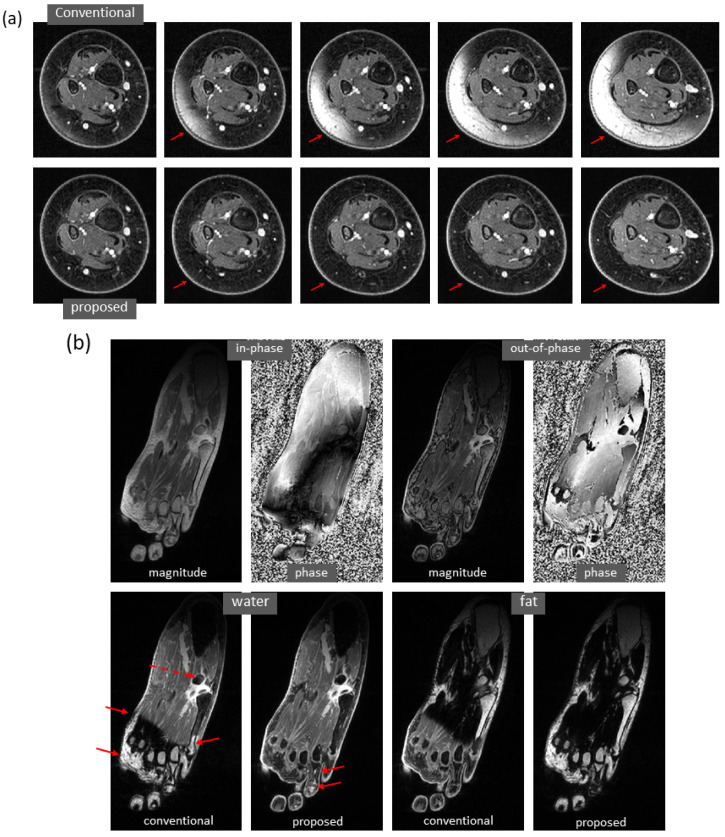

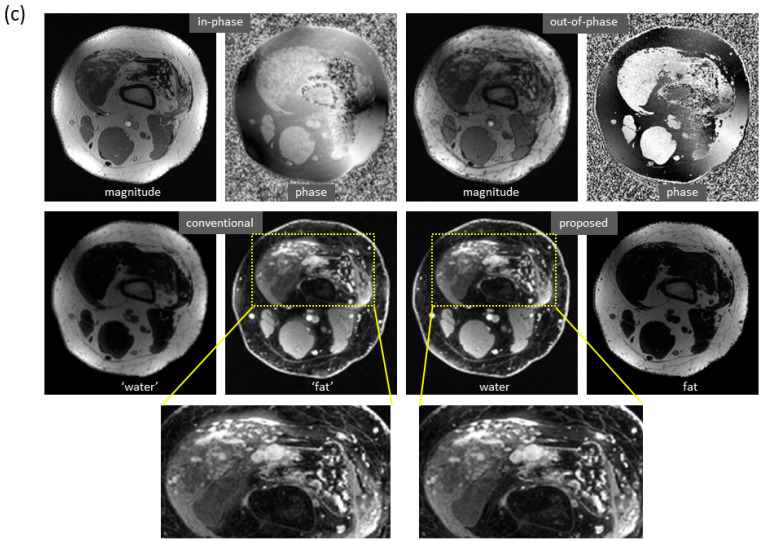
Marked correction of severe water/fat swaps. (**a**) In an examination of the ankle, progressively severe water/fat swaps occurred in the reference images as they moved farther from isocenter (every ninth slice is shown). In the predicted images, the water/fat swaps were completely corrected. (**b**) In an examination of the foot, severe water/fat swaps appeared in the peripheral region of the reference images, where the dashed arrow showed an accessory ossicle with marrow edema that also had a local swap in its marrow. The water/fat swaps were substantially compensated in the predicted images, which only had smaller regions of swaps (solid arrows). (**c**) Global water/fat swaps, which occurred in the reference image, were corrected using the proposed deep learning method. An improved spatial resolution was observed in the predicted image.

More examples with severe local water/fat swaps as well as global water/fat swaps are shown in Figure 5. In an ankle examination (Figure 5a), as the slices moved farther from isocenter, progressively severe water/fat swaps occurred in the peripheral region of reference images; the water/fat swaps were corrected in the predicted images. In a foot examination (Figure 5b), severe water/fat swaps appeared in the reference images (the dashed arrow pointed to an accessory ossicle with edema, and a local swap appeared in its marrow); the predicted images only had a few smaller swaps. In a knee study (Figure 5c), global water/fat swaps occurred, which in fact was not uncommon in conventional water–fat separation. Using the proposed method, global water/fat swaps were eliminated, and a slightly improved spatial resolution was observed, which can be attributed to the use of convolution for upsampling (expected to outperform conventional upsampling processing).

**Figure 6:**
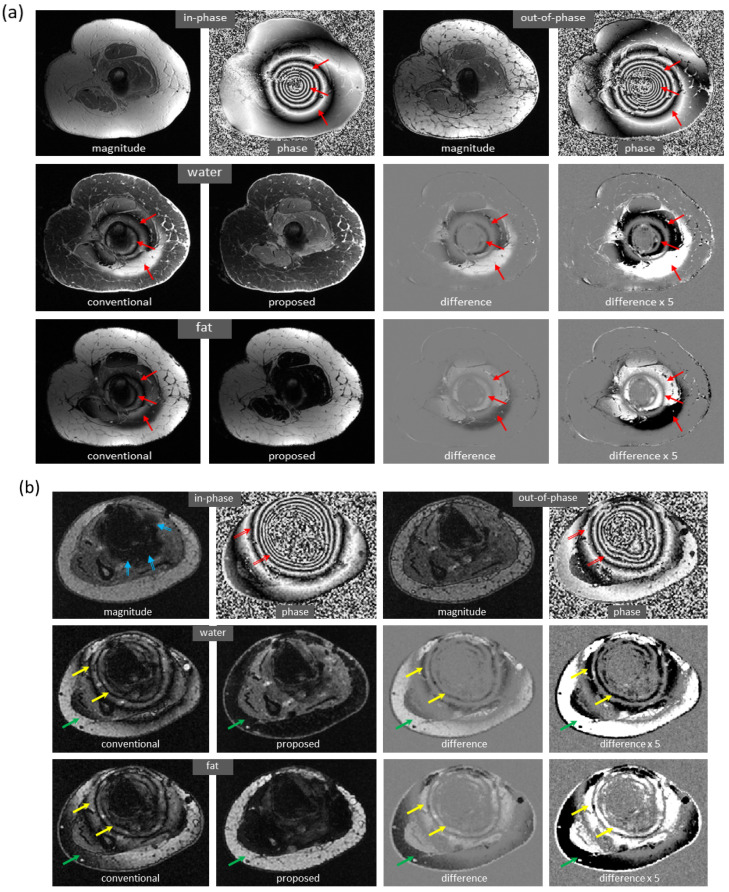
Marked reduction of metal-induced artifacts, where the training set lacked any examples with metallic implants. (**a**) In this knee case acquired on a PET-MRI scanner, severe off-resonance artifacts occurred in the reference images since the local magnetic field near metal changed dramatically (as can be seen from the input phase images). In the predicted images, metal-induced artifacts were largely corrected. (**b**) In another ankle case acquired on a 1.5T MRI scanner, signal loss (blue arrow) and phase corruption (red arrow) were observed in the input images, resulting in artifacts in the reference images (yellow arrow). In addition, severe water/fat swaps (green arrow) occurred in the peripheral region of this off-isocenter slice. Both metal artifacts and water/fat swaps were mitigated in the predicted images, even when the model was trained with only knee images acquired on 3T scanners.

Figure 6 shows the mitigation of severe artifacts induced by a metallic object, further demonstrating robustness to out-of-distribution data. The dramatic changes in local magnetic fields made the cases with metallic implants very challenging. In a knee study (Figure 6a), phase corruption in dual-echo images caused severe artifacts in the reference water/fat images. Interestingly, the predicted images lacked these artifacts. Here, the test images were acquired on a PET-MRI scanner (whereas the majority of training data were obtained on MRI scanners). In an ankle case (Figure 6b), both signal loss and phase corruption were observed in dual-echo images, leading to artifacts in the reference images. The metal artifacts, along with additional water/fat swaps that occurred in peripheral region of off-isocenter slices, were corrected in the predicted images even though the ankle images were acquired on a 1.5T MRI scanner (while training data were obtained on 3T scanners). Of note, for these two cases, is the fact that the training sets lacked any examples with metallic implants.

**Figure 7:**
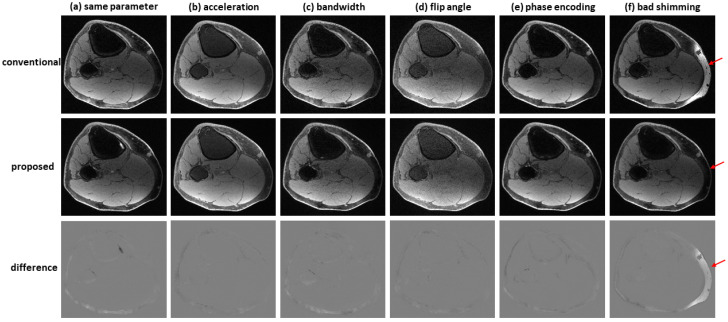
Water–fat separation from non-contrast-enhanced dual-echo images using a model trained with contrast-enhanced images. The non-contrast-enhanced dual-echo images were acquired using (**a**) the same imaging parameters as those adopted in contrast-enhanced studies, (**b**) an acceleration factor of 2, (**c**) bandwidth of 83.3 kHz, (**d**) flip angle of 25°, and (**e**) phase encoding of 224. In these cases, the predicted images had high fidelity to the reference images. (**f**) When bad shimming was intentionally imposed, water/fat swaps that occurred in the reference image were corrected in the predicted image.

Figure 7 shows an example of water–fat separation from non-contrast-enhanced dual-echo images using a model trained with contrast-enhanced images. Even if the imaging parameters were slightly different from those adopted in contrast-enhanced studies (with an acceleration factor of 2, bandwidth of 83.3 kHz, flip angle of 25°, or phase encoding of 224), the predicted images had high fidelity to the reference images, indicating the proposed method has high potential to support flexible imaging parameters. When bad shimming was intentionally imposed (to generate highly inhomogeneous $B_{0}$ field), water/fat swaps that occurred in reference images were corrected in the predicted images.

## 4. Discussion

In this study, we developed a deep learning-based dual-echo water–fat separation approach that is more practically useful than previous approaches. Dual-echo water–fat separation is highly desirable in clinical practice due to the high data acquisition efficiency and the common clinical use of dual-echo imaging. In anatomic regions where dual-echo imaging is an essential part of the clinical imaging protocol, no additional acquisition is needed for the proposed water–fat separation method. Furthermore, the marked reduction in compute time is critical for contrast-enhanced imaging, since technologists must rapidly evaluate the images for adequacy.

In addition, the proposed deep learning method offers improved robustness over conventional water–fat separation approaches. With a large number of network parameters, hierarchical network architecture, and densely connected shortcuts, the deep neural network can effectively provide complicated nonlinear mapping for water–fat separation as well as for other image-to-image translation problems [28,29,30,31,32]. The deep learning model takes a variety of contributing factors into consideration and accomplish the task of water–fat separation in a series of steps. Using the proposed model, the proposed method can largely compensate for local water/fat swaps and eliminate global water/fat swaps.

Of particular interest is the fact that metal-induced artifacts have been substantially corrected using the proposed method. This is likely attributed to an accurate estimation of the $B_{0}$ map. The capability of deep learning to estimate $B_{0}$ map from dual-echo images was confirmed in our previous liver study [30]. The deep learning-based $B_{0}$ estimation, as we believe, not only relies on the smoothly varying the characteristics of magnetic field homogeneity (as conventional approaches do) but also utilizes other a priori information extracted by the deep learning model. In fact, deep learning has the capability to integrate a variety of a priori knowledge. For the same reason, the proposed method is robust to background noise. It was found that prediction models established using original unsegmented images had a very similar performance to those trained using segmented images (with non-tissue background removed), indicating a mask was implicitly produced from the magnitude of input images and used in combination with other information.

In this study, we used the projected power method, a state-of-the-art dual-echo water–fat separation technique, to generate reference images. While other influential water–fat separation techniques (e.g., VARPRO, IDEAL) can potentially be used to produce reference images, the establishment and validation of the prediction models will be difficult due to the extra multi-echo scans required by reference images.

To fully support flexible imaging parameters, we proposed a mechanism that takes imaging parameters as additional network input. In this way, a priori knowledge (e.g., of echo times) is not implicitly learned in training but rather explicitly given and, as a result, water–fat separation is improved. With this mechanism, it is possible to use even more flexible echo times or other imaging parameters for data acquisition. In general, the mechanism of including imaging parameter values as additional network input can be adopted in other applications, such as quantitative MRI.

We also evaluated the generalization capabilities of the proposed method. Models trained with only knee images performed well on diverse data sets acquired on various MRI or PET-MRI scanners. Furthermore, promising results have been obtained in a preliminary non-contrast-enhanced experiment, where relatively accurate water/fat images were derived from non-contrast-enhanced images using models trained with only contrast-enhanced images. More investigation is needed, but this is important because non-contrast-enhanced studies facilitate the evaluation of the model’s capability to support flexible imaging parameters. It is of note that contrast-enhanced dual-echo imaging was used for water–fat separation in this study due to its clinical significance and wide application in cancer management.

The study has some limitations. Most data sets were contrast-enhanced images acquired from pediatric patients. The proposed method should be further validated with additional data sets: both contrast and non-contrast-enhanced, other anatomic regions, pediatric and adult patients, and images acquired using even more flexible imaging parameters (such as echo time).

Because of the similarity in resolving phase ambiguity, the proposed deep learning approach can potentially be used to solve other problems, such as velocity unwrapping in phase contrast MRI [42] and phase-sensitive inversion-recovery reconstruction [43].

## 5. Conclusions

A deep learning method was developed for fast water–fat separation from dual-echo chemical shift-encoded images, and robustness to several forms of out-of-distribution data was demonstrated.

## Data Availability

The original data and source code will be available publicly upon publication of the manuscript.
